# Dosimetric Comparison of Hypofractionated Multi-Beam Intensity-Modulated Radiation Therapy and Volumetric Modulated Arc Therapy With Flattened Beam and Flattening-Filter-Free Beam for Skull Base Meningioma Adjacent to Optic Pathways

**DOI:** 10.7759/cureus.8690

**Published:** 2020-06-18

**Authors:** Ikue Isobe, Yoshimasa Mori, Naoki Kaneda, Chisa Hashizume, Tsuneo Ishiguchi, Kojiro Suzuki

**Affiliations:** 1 Radiology, Aichi Medical University, Nagakute, JPN; 2 Radiation Oncology and Neurological Surgery, Shin-Yurigaoka General Hospital, Kawasaki, JPN; 3 Radiology and Radiation Oncology, Aichi Medical University, Nagakute, JPN; 4 Neurological Surgery, Ookuma Hospital, Nagoya, JPN; 5 Neurological Surgery, Aoyama General Hospital, Toyokawa, JPN; 6 Radiation Oncology, Nagoya Kyoritsu Hospital, Nagoya, JPN

**Keywords:** skull base, optic nerve, intensity-modulated radiation therapy, volumetric-modulated arc therapy, flattening filter free

## Abstract

Background

Since the optic pathways are the most vulnerable to radiation, the treatment of skull base tumors involving them is challenging. In this study simulation plans by multi-beam (MB) intensity-modulated radiation therapy (IMRT) and volumetric modulated arc therapy (VMAT), both with the flattened beam (FB) and flattening-filter-free beam (FFF), were compared in terms of covering of the target and sparing of the optic pathways.

Materials and methods

Treatment planning was simulated by MB-IMRT with FB and FFF and by 2-rotational VMAT with FB and FFF in three cases of skull base meningioma [volume of the planned target volume (PTV; PTV margin=2 mm except for overlapping area with optic pathways or brainstem): 8.6 ml, 34.6 ml, and 55.3 ml respectively], which were treated previously by multi-fractionated MB-IMRT [45 Gy/18 fx. (fraction) with 7-, 6-, and 5-beam] using a conventional Novalis (BrainLAB, Tokyo, Japan) planned by iPlan (BrainLAB, Tokyo, Japan). In all three cases, the optic pathways were adjacent to the lesion. The reference CT with contouring data set of target volumes [gross tumor volume (GTV) and PTV] and OARs (organs at risk) was transferred from iPlan to Eclipse (Varian Medical Systems, Tokyo, Japan). In this study, hypofractionated radiation therapy by 30 Gy/5 fx. was designed; 95% dose (28.5 Gy/5 fx.) was prescribed to D95 (dose to 95% volume of PTV). Conformity index (CI), homogeneity index (HI, D5/D95), D[0.1 ml] (dose to 0.1 ml) for optic pathways, and D[1 ml] for brainstem and eyes, and V[20 Gy] (volume delivered with 20 Gy or more/5 fx.) of the whole brain were evaluated.

Results

The indices did not differ between FB and FFF, in either MB-IMRT or VMAT. Between MB-IMRT and VMAT, the indices were similar. The mean dose of PTV and HI was a little larger with MB-IMRT than with VMAT. D[0.1 ml] of the optic pathways and D[1 ml] of the ipsilateral eye were smaller with VMAT in all three cases. D[1 ml] of the brainstem was smaller with VMAT in two cases, though it was similar in one case.

Conclusion

Based on our findings, VMAT with FFF might be the optimal method to treat cases of skull base meningioma involving optic pathways. However, further studies involving more cases are required to arrive at a conclusive verdict.

## Introduction

Stereotactic radiosurgery (SRS)/stereotactic radiotherapy (SRT) is an effective and safe option for the treatment of benign brain tumors if the tumor is not large [1-5]. Whether SRS/SRT can be performed safely depends on the desired dose given to the tumor margin and, simultaneously, an acceptable dose to the organs at risk (OARs) such as the optic pathways and brainstem. As the optic pathways are thought to be the most vulnerable to irradiation, benign skull base tumors involving them are challenging to treat. In the clinical situation, when deciding how best to treat skull base benign tumors, we usually devise some different plans, such as dynamic conformal multi-arc therapy and intensity-modulated radiation therapy (IMRT). Then we choose the best of these, namely the most effective and safest one. For complex shape and proximity of OARs to the target tumor, the IMRT technique has a major advantage in reducing the dose to the OARs. Recently, besides static gantry-angle multi-beam (MB)-IMRT, volumetric modulated arc therapy (VMAT) has also become available. In MB-IMRT planning, optimal gantry angle selection by well-practiced planners is necessary. On the other hand, the VMAT optimization process includes optimization of intensity from a continuous surrounding arc beam. In addition, the flattening-filter-free (FFF) beam has become available recently, which is good for SRS/SRT with a large single fraction dose. Treatment with the FFF beam has the advantage of shorter treatment time, compared to that with the flattened beam (FB), due to a higher dose rate and lower exposure dose at the surrounding normal structures, especially when the radiation field is large [6]. In this study, simulation plans by MB-IMRT and VMAT, both with FB and FFF, were compared in terms of covering of target and sparing of OARs, especially the optic pathways.

## Materials and methods

Treatment planning was simulated by coplanar MB-IMRT with FB and FFF and by 2-coplanar rotational VMAT with FB and FFF in three cases (Table 1) of skull base meningioma [volume of the planned target volume (PTV; PTV margin=2 mm except for overlapping area with optic pathways or brainstem): 8.6 ml, 34.6 ml, and 55.3 ml respectively], which were treated previously by multi-fractionated MB-IMRT [45 Gy/18 fx. (fraction) with 7-, 6-, and 5-beam] using a conventional Novalis (BrainLAB, Tokyo, Japan) planned by iPlan version 4.1.2 (BrainLAB, Tokyo, Japan) workstation in Nagoya Radiosurgery Center, Nagoya Kyoritsu Hospital from July 2011 through April 2014.

**Table 1:** Characteristics of the tumors and original clinical treatments *Visual field (VF) defect: left upper homonymous quadrant anopsia. **Central scotoma in the left eye PTV: planning target volume; MB-IMRT: multi-beam intensity-modulated radiation therapy; fx.: fraction

Case	PTV (ml)	Tumor location	Visual status	MB-IMRT	Dose and fractions	Prior therapy
1	8.6	Right ant. clinoid	VF defect*	7 beams	45 Gy/18 fx.	surgical resection
2	34.6	Left sphenoid ridge	Scotoma**	6 beams	47.5 Gy/19 fx.	Surgical resection
3	55.3	Right sphenoid ridge	intact	5 beams	47.5 Gy/19 fx.	Surgical resection

**Figure 1:**
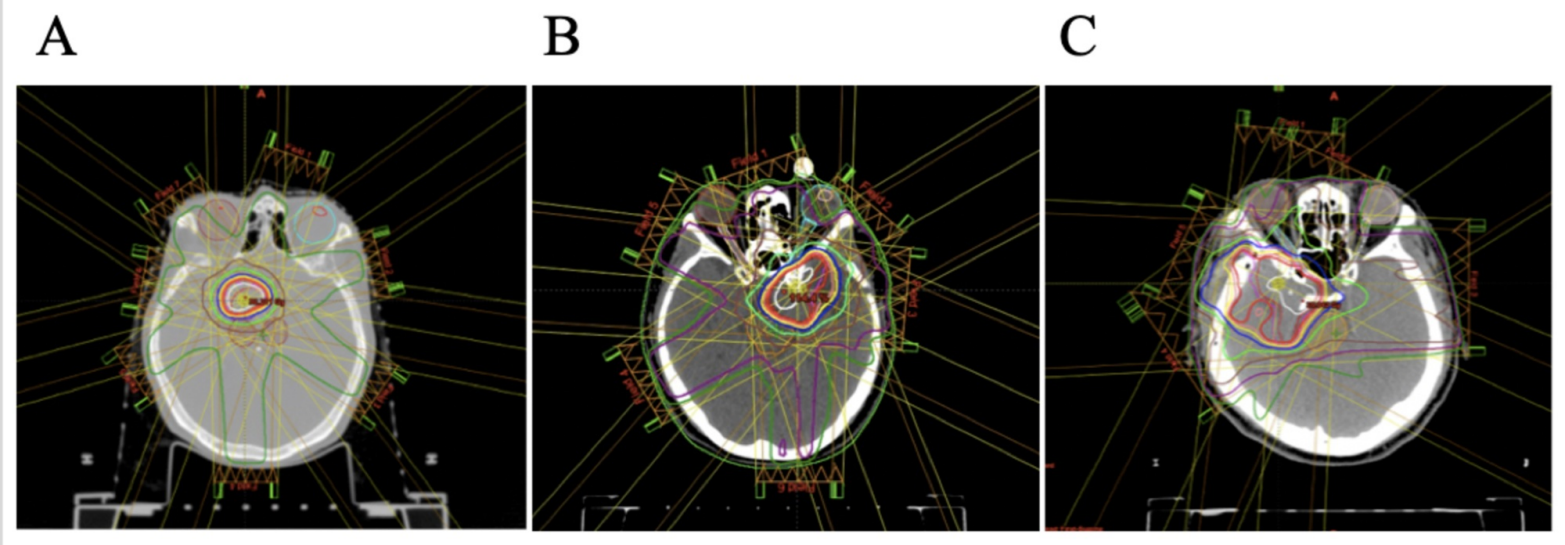
Axial images showing coplanar beam angle and dose distribution in three cases A: Case 1; B: Case 2; C: Case 3

In all three cases, the optic pathways were adjacent to the lesion. The reference CT with contouring data set of target volumes [gross tumor volume (GTV) and PTV] and OARs was transferred from iPlan to Eclipse (equipped with Acuros XB version 11.0.31, Varian Medical Systems, Tokyo, Japan) workstation. In this study, hypofractionated radiation therapy by 30 Gy/5 fx. was designed; 95% dose (28.5 Gy/5 fx.) was prescribed to D95% (dose to 95% volume of PTV). The same beam angles for MB-IMRT were employed in each case as those had been clinically used during Novalis multi-fractionated MB-IMRT (Figure 1) as a result of trial-and-error to make a better plan.

Conformity index (CI), homogeneity index (HI), gradient index (GI), D[0.1 ml] (dose to 0.1 ml) for optic pathways, and D[1 ml] for brainstem and eyes, and V[20 Gy] (volume delivered with 20 Gy or more/5 fx.) of the whole brain were evaluated. The Research Ethics Board of Aichi Medical University (2015-H333) and the Clinical Research Committee of Nagoya Kyoritsu Hospital (K83-02) approved this study. Informed consent was waived.

Imaging protocol

The treatment-planning images were acquired, during original Novalis treatment planning, with 4-detector CT (Light Speed Plus; GE Healthcare, Tokyo, Japan) and MRI using a 1.5-Tesla or 3.0-Tesla scanner (Signa Echo Speed Plus 1.5 T, Signa HDxt 3.0 T; GE Healthcare, Tokyo, Japan). The references for dose calculation in treatment planning were the CT images. CT image resolution of 512 × 512 pixels in the axial plane and a slice thickness of 1.25 mm were adopted. To determine GTV [=CTV (clinical target volume)], contrast-enhanced CT and MRI were acquired. Conditions for non-contrast and contrast-enhanced CT were the same except for the size of the field of view. The slice thickness of MRI was specified as 1-2 mm, depending on the tumor size, by 3-dimensional (3D)-spoiled gradient-recalled acquisition in the steady-state sequence with gadolinium enhancement and 3D fast spin echo.

Eclipse treatment planning

MB-IMRT with FFF and FB and VMAT with FFF and FB plans for TrueBeam STx version 2 (Varian Medical Systems, Tokyo, Japan) were made. Parameter evaluation of the dose-volume histogram (DVH) was performed considering target coverage and the dose limitation for OARs. The dose constraints for OARs used in the IMRT optimization process were determined according to the tolerance dose; e.g., 55 Gy for D[0.1 ml] of the brainstem, 50 Gy for D[0.1 ml] of optic nerves, 10 Gy for D[maximum], and 50 Gy for D[1 ml] of eyes in a 2 Gy per fx. regime. In Case 2 and Case 3, 50 Gy for D[0.1 ml] of the acoustic nerve was also added. The prescription dose to PTV D95 was 28.5 Gy/5 fx. (95% dose).

Dosimetric analysis

The DVH analyses were made with the indices shown below:

The CI was defined as the Radiation Therapy Oncology Group (RTOG) CI = PIV/PTV [7], where the volume of the prescription isodose volume (PIV) was divided by the PTV. The HI was calculated with the formula: dose HI (DHI) = D95% (prescribed dose)/Dmax (maximum dose) and moderate dose HI (mDHI) = D95%/D5% [8].

The dose of OARs was evaluated with the indices as follows:

D[0.1 ml] for the optic pathways, D[1 ml] for the eyes and the brainstem, and V[20 Gy] (volume receiving 20 Gy) of the whole brain.

The brainstem was contoured manually as high as the midbrain. The whole brain was contoured, at first, in two parts. In the lower axial slices, as high as at the top of the contoured brainstem, the cerebellum and the cerebral lobes were contoured without including the subarachnoid cisterns. In the upper axial slices, intra-dural spaces were contoured as the brain. Then the whole brain was made by the accumulation of these and the brainstem [9].

## Results

The indices of all 12 simulation plans are summarized in Table 2.

**Table 2:** Summary of dose-volume histogram analysis for PTV, GTV, and organs at risk in three cases *mDHI = D[5%]/D[95%]. **CI = (V[95% dose] of the body)/(V[95% dose] of the PTV) VMAT: volumetric modulated arc therapy; MB-IMRT: multi-beam intensity-modulated radiation therapy; FFF: flattening-filter-free beam; FB: flattened beam; PTV: planning target volume; GTV: gross tumor volume; D95: dose prescribed at 95% volume; D99: dose at 99%; D[0.1 ml]: dose at 0.1 ml; D[1 ml]: dose at 1 ml; V[20 Gy]: volume of 20 Gy or more

Case 1				
	VMAT_FFF	VMAT_FB	MB-IMRT_FFF	MB-IMRT_FB
PTV (8.6 ml)				
D95 (Gy)	28.5	28.5	28.5	28.5
Mean dose (Gy)	30.3	30.3	33.1	33.1
mDHI*	1.10	1.10	1.27	1.27
CI**	1.90	1.98	1.91	1.91
GTV (4.2 ml)				
D99 (Gy)	28.9	29.0	29.3	29.3
Optic nerves and tracts (1.86 ml)				
D[0.1 ml] (Gy)	24.1	24.0	24.8	25.4
Ipsilateral eye (9.80 ml)				
D[1 ml] (Gy)	5.9	6.4	7.3	7.1
Contralateral eye (9.80 ml)				
D[1 ml] (Gy)	5.4	5.2	3.4	3.6
Brainstem (25.5 ml)				
D[1 ml] (Gy)	15.7	16.2	15.6	15.7
Brain (1083.3 ml)				
V[20 Gy] (ml)	11.2	12.1	9.6	10.3

Case 2				
	VMAT_FFF	VMAT_FB	MB-IMRT_FFF	MB-IMRT_FB
PTV (34.6 ml)				
D95 (Gy)	28.5	28.5	28.5	28.5
Mean dose (Gy)	30.21	30.36	31.71	31.8
mDHI*	1.11	1.10	1.19	1.19
CI**	1.26	1.27	1.24	1.25
GTV (22.3 ml)				
D99 (Gy)	28.0	27.4	26.9	26.8
Optic nerves and tracts (2.40 ml)				
D[0.1 ml] (Gy)	21.3	20.8	22.9	22.9
Ipsilateral eye (9.27 ml)				
D[1 ml] (Gy)	7.8	8.1	9.4	9.5
Contralateral eye (9.86 ml)				
D[1 ml] (Gy)	6.3	7.0	8.1	7.8
Brainstem (27.8 ml)				
D[1 ml] (Gy)	20.3	20.8	23.7	23.9
Brain (1161.0 ml)				
V[20 Gy] (ml)	16.8	17.0	16.5	16.4

Case 3				
	VMAT_FFF	VMAT_FB	MB-IMRT_FFF	MB-IMRT_FB
PTV (55.3 ml)				
D95 (Gy)	28.5	28.5	28.5	28.5
Mean dose (Gy)	30.24	30.27	32.58	32.67
mDHI*	1.11	1.11	1.27	1.28
CI**	1.26	1.23	1.57	1.57
GTV (33.6 ml)				
D99 (Gy)	28.6	28.4	27.7	27.6
Optic nerves and tracts (2.86 ml)				
D[0.1 ml] (Gy)	22.1	22.2	26.0	25.9
Ipsilateral eye (11.36 ml)				
D[1 ml] (Gy)	9.8	9.9	14.0	13.9
Contralateral eye (11.02 ml)				
D[1 ml] (Gy)	6.8	6.2	4.9	5.0
Brainstem (27.7 ml)				
D[1 ml] (Gy)	20.3	20.2	23.5	23.6
Brain (1257.8 ml)				
V[20 Gy] (ml)	26.6	27.0	44.0	44.9

**Figure 2:**
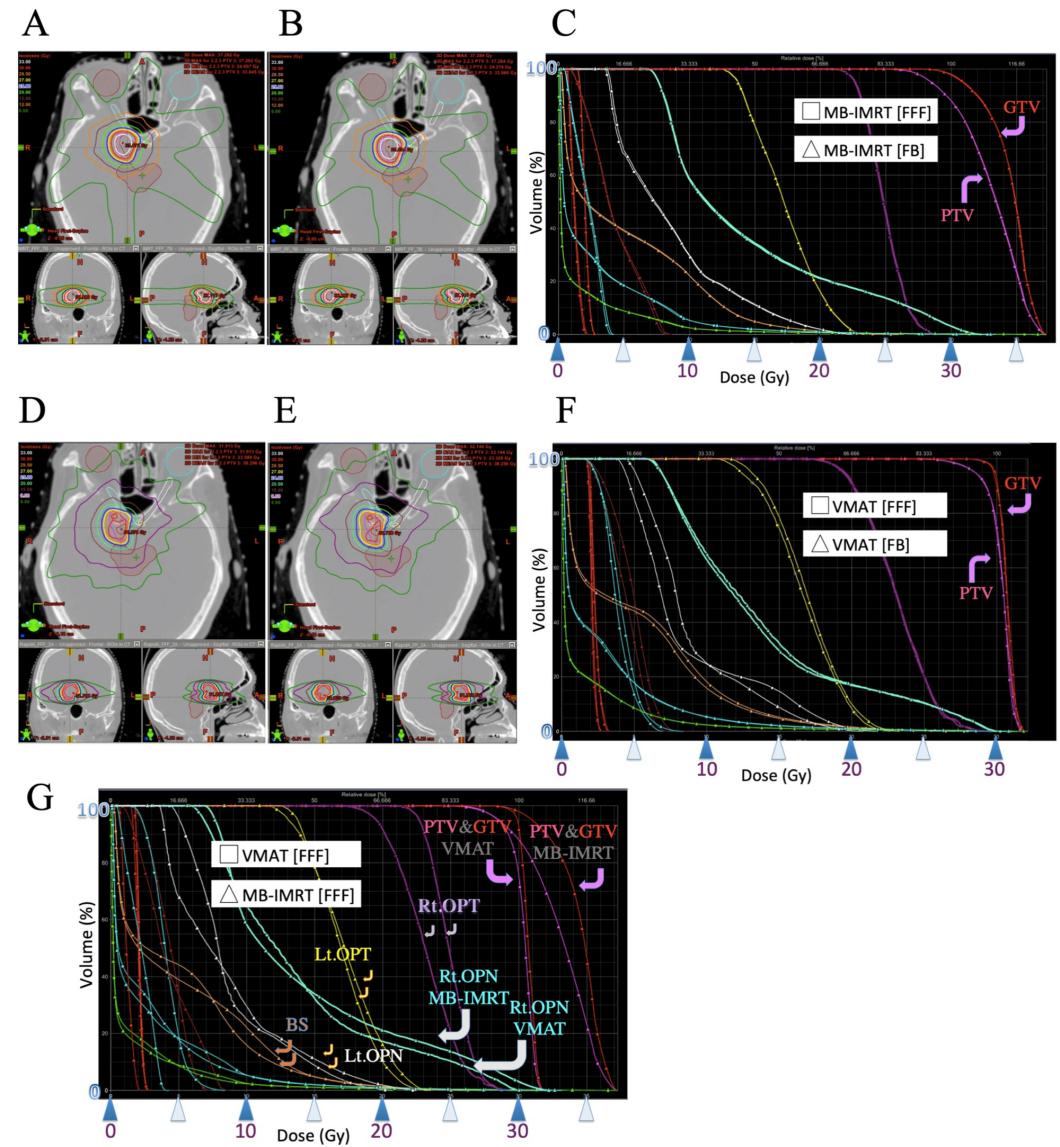
Case 1 Images showing dose distribution of MB-IMRT with FFF (A), MB-IMRT with FB (B), VMAT with FFF (D), and VMAT with FB (E). Dose-volume histogram (DVH) of MB-IMRT with FFF and MB-IMRT with FB were very similar (C). DVHs of VMAT with FFF and VMAT with FB were also very similar (F). However, there were some differences between the DVH of VMAT with FFF and that of MB-IMRT with FFF (G) MB-IMRT: multi-beam intensity-modulated radiotherapy; FFF: flattening-filter-free beam; VMAT: volumetric modulated radiotherapy; FB: flattened beam; DVH: dose-volume histogram; PTV: planning target volume; GTV: gross tumor volume; Rt.: right; Lt.: left; OPN: right optic nerve; OPT: optic tract; BS: brainstem

The indices were similar between with FB and with FFF, in both MB-IMRT and VMAT in all three cases as shown in Table 2. In addition, Figure 2A, 2B, 2C (MB-IMRT), and Figure 2D, 2E, 2F (VMAT) show that dose distribution and DVH were similar between FB and FFF, in both MB-IMRT and VMAT in Case 1 (Figure 2). 

**Figure 3:**
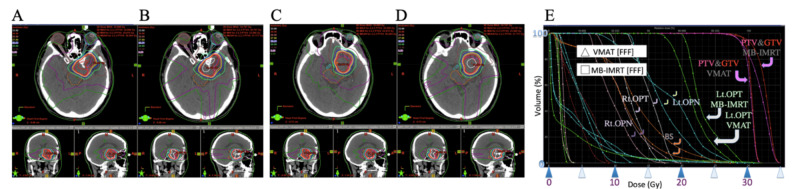
Case 2 Images showing dose distribution of VMAT with FFF (A, C) and MB-IMRT with FFF (B, D). C and D show more upper axial images than A and B. There were some differences between the DVH of VMAT with FFF and that of MB-IMRT with FFF (E) MB-IMRT: multi-beam intensity-modulated radiotherapy; FFF: flattening-filter-free beam: VMAT: volumetric modulated radiotherapy; FB: flattened beam; DVH: dose-volume histogram; PTV: planning target volume; GTV: gross tumor volume; Rt.: right; Lt.: left; OPN: right optic nerve; OPT: optic tract; BS: brainstem

**Figure 4:**
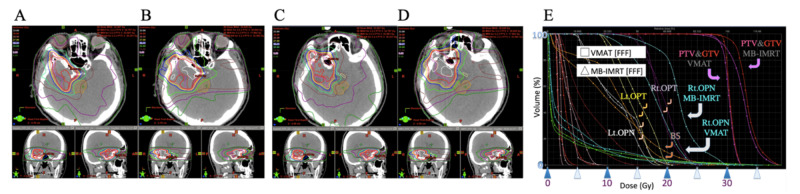
Case 3 Images showing dose distribution of VMAT with FFF (A, C) and MB-IMRT with FFF (B, D). C and D show more upper axial images than A and B. There were some differences between the DVH (E) and the dose distribution of VMAT with FFF and those of MB-IMRT with FFF MB-IMRT: multi-beam intensity-modulated radiotherapy; FFF: flattening-filter-free beam: VMAT: volumetric modulated radiotherapy; FB: flattened beam; DVH: dose-volume histogram; PTV: planning target volume; GTV: gross tumor volume; Rt.: right; Lt.: left; OPN: right optic nerve; OPT: optic tract; BS: brainstem

On the other hand, certain tendencies in some of the indices between VMAT and MB-IMRT in all three cases were observed. Table 2 showed the indices. Figure 2A, 2D, 2G (Case 1), Figure 3 (Case 2), and Figure 4 (Case 3) show dose distribution and DVHs.

The mean doses of PTV and mDHI were a little larger in MB-IMRT than in VMAT in all three cases, and both showed a slightly higher dose inside the PTV. The CI was similar in Case 1 and Case 2 but it was larger in MB-IMRT in Case 3. D99 of GTV was similar in all three cases. D[0.1 ml] of the optic nerves and tracts and D[1 ml] of the ipsilateral eye were less in VMAT than in MB-IMRT in all three cases. D[1 ml] of the contralateral eye was less with MB-IMRT than in VMAT in Case 1 and Case 3 but more in Case 2. D[1 ml] of the brainstem was similar in Case 1 but less in VMAT than with MB-IMRT in Case 2 and Case 3. V[20 Gy] of the brain was similar in Case 1 and Case 2 but much larger in MB-IMRT than in VMAT in Case 3.

## Discussion

Removal of the flattening filter will result in an FFF beam with a conical shaped fluence distribution, an increase in dose rate, and a reduction in head scatter that may result in shorter treatment times and beam-on times as well as a lower peripheral dose that may increase the possibility of sparing OARs in a large radiation field [6]. In all three of the present cases, sparing of OAR including the brain was similar between both FFF and FB, perhaps because the radiation fields were not wide. However, shorter treatment time would be a merit of FFF.

VMAT is a technique for delivering conformal dose distributions that consist of IMRT delivered continuously as the gantry rotates around the patient in arcs. In MB-IMRT planning, optimal beam angle selection with a trial-and-error manner by well-practiced planners might be necessary. In VMAT, the planning process includes optimization of the gantry rotation speed, multi-leaf collimator field aperture, and the dose rate. Comparisons between MB-IMRT and VMAT have been reported in some studies confirming the potential of treatment time reduction in VMAT and similar or better sparing of OARs without compromising on the dose to target [6,10]. In our study of skull base meningiomas adjacent to optic pathways, VMAT had advantages in sparing of the OAR including the optic pathways and ipsilateral eye, though the results of only three cases were documented.

## Conclusions

In our study to evaluate simulation plans of skull base meningiomas adjacent to optic pathways, FB and FFF both in VMAT and in MB-IMRT were similar in terms of target covering and OAR sparing. However, FFF had the advantage of reducing the treatment time. Dose to the optic pathways and ipsilateral eye tended to be a little less in VMAT than MB-IMRT. According to this study, VMAT with FFF might be the best method to treat cases of skull base meningioma involving optic pathways, though further investigations involving more cases are necessary to reach a conclusive decision.
